# Fungal sepsis in a 7-month-old female: diagnosis through peripheral blood smear

**DOI:** 10.1016/j.mmcr.2025.100708

**Published:** 2025-05-22

**Authors:** Yihun Bedaso, Tadesse Alemayehu, Amanuel Anegagregn Bizuneh, Dereje Zeleke Haile, Seble Seifu Zeleke, Mekdes Shifeta, Agete Tadewos Hirigo

**Affiliations:** a Hawassa University Comprehensive Specialized Hospital, Hawassa, Ethiopia; b School of Medicine, College of Medicine and Health Science, Hawassa University, Hawassa, Ethiopia; c School of Medical Laboratory Science, College of Medicine and Health Science, Hawassa University, Hawassa, Ethiopia

**Keywords:** Fever, Peripheral blood smear, Yeast cells, Fungal sepsis

## Abstract

We report a case of a 7-month-old female with prolonged high-grade fever unresponsive to broad-spectrum antibiotics and antimalarial drugs. Despite negative cultures and imaging, a Giemsa-stained peripheral blood smear on day 28 revealed yeast cells, suggesting fungal sepsis in the context of prolonged antibiotic exposure and thrombocytopenia. The patient responded well to oral fluconazole, with full clinical recovery. This case underscores the value of peripheral blood smear examination in diagnosing yeast infections in resource-limited settings.

## 1 Introduction

Fungemia caused by *Candida species (spp)*, remains the most common form of candidemia and is a leading cause of morbidity and mortality among healthcare-associated bloodstream infections. In addition, although *Cryptococcus* and *Trichosporon* species can cause fungemia, such infections are rare compared to *Candida* spp [1]. Fungal pathogens, including *Candida*, have become significant contributors to nosocomial bloodstream infections [2]. The infection often becomes apparent days or weeks after hospitalization, with common risk factors including recent abdominal surgery, prolonged hospital or intensive care unit stay, haemodialysis, organ transplantation, malignancy, broad-spectrum antibiotic use, total parenteral nutrition, and central venous catheter placement [3,4]. Although less common than candidemia, endemic mycoses caused by *Blastomyces*, *Histoplasma*, *Paracoccidioides*, and *Coccidioides* have been implicated in severe systemic infections, especially among immunocompromised patients. [5]. *Candidemia* represents a significant public health concern due to its association with poor patient outcomes, prolonged hospitalizations, increased healthcare costs, and an attributable mortality rate ranging from 35 % to 70 % [6].

The quantity of yeast infection seen in such case is rare, making the detection of yeast cells through blood smear review unlikely in most instances. We present the case of a 7-month-old female with intermittent high-grade fever, initially referred as a fever of unknown origin in whom yeast cells were identified in the peripheral blood smear.

## 2 Case report

A 7-month-old female was admitted to a paediatric specialty clinic with intermittent high-grade fever. She was treated with ampicillin and ceftriaxone for 10 days. However, the cause remained unclear, and she was subsequently referred to Hawassa University Comprehensive Specialized Hospital for further evaluation as a case of fever of unknown origin. The patient presented to the pediatric emergency department with intermittent, non-whooping, non-barking cough and high-grade fever lasting for two weeks. Physical examination was unremarkable, and investigations were normal, except for a diagnosis of pneumonia with rickets. She was appropriately vaccinated for her age and had no known contact with tuberculosis (TB) patients or individuals with chronic cough. She also showed no signs of night sweats, weight loss, diaphoresis or feeding difficulties.

On admission (day 0), her complete blood count revealed a haemoglobin (Hb) level of 12.1 g/dl (normal range: 12.5–16.3 g/dl), a white blood cell (WBC) count of 11.35 × 10^3^/μl (normal range: 5.0–19.0 × 10^3^/μl), and a platelet count of 425 × 10^3^/μl (normal range: 150–450 × 10^3^/μl) with a marked increase in the absolute lymphocyte count of 7.63 × 10^3^/μl. Her serum electrolytes were normal, and the Gene Xpert result for tuberculosis (TB) was negative. However, the blood film (BF) revealed *Plasmodium vivax.* Following this her blood culture showed coagulase-negative staphylococci (CoNS), which are commensal bacteria typically encountered in hospital settings and considered contaminants.

She was initially assessed for severe pneumonia, rickets and malaria and was treated with Vancomycin, Ceftazidime, Artesunate and Vitamin D, but showed no improvement. Four days later, she developed vomiting, diarrhoea, and persistent fever. As a result, the antibiotics were adjusted to Vancomycin and Ciprofloxacin but there was still no improvement. On the fifth day, the antibiotics were further revised to Vancomycin and Cefepime injections, and subsequently changed to Meropenem injection monotherapy.

Despite ongoing treatment, on the 15th day after admission, the patient's condition worsened with increased vomiting, diarrhoea, cough and respiratory distress, resulting in oxygen desaturation to 82 %. However, diagnostic tests including echocardiogram (ECHO), abdominal ultrasound, brain CT scan, erythrocyte sedimentation rate (ESR), urinalysis, and Gene Xpert for tuberculosis all revealing normal results, and there was no growth in the repeated blood or stool cultures. Despite the negative results for tuberculosis, the fever, vomiting, diarrhoea and cough continued to progress without improvement. As a result, anti-TB treatment with pyridoxine was initiated as a precautionary measure. The regimen included rifampicin, isoniazid, pyrazinamide, and ethambutol, along with pyridoxine, and was continued for two months.

**Fig. 1:**
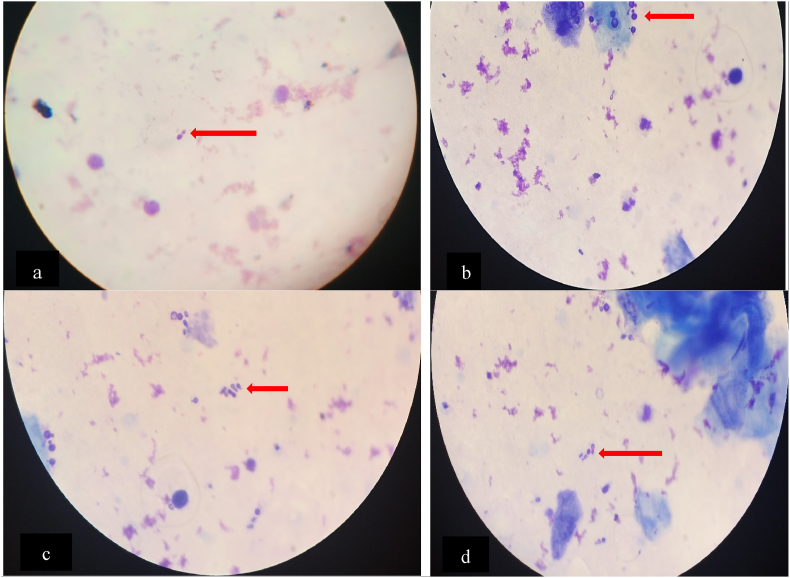
(a, b, c, d) Peripheral thick blood smear displaying yeast cells (Giemsa stain, 1000x magnification).

On the 18th day of admission, due to lack of progress, tests including a CBC, BF, cerebrospinal fluid (CSF) analysis and renal function tests (RFT) were repeated. The CBC revealed a haemoglobin level of 9.3 g/dL, a white blood cell count of 5.83 × 10^3^/μL, and a platelet count of 46 × 10^3^/μL (indicating thrombocytopenia), with a markedly elevated absolute lymphocyte count of 3.85 × 10^3^/μL from the total WBC count. Both the CSF and RFT results were also normal. Microscopic examination of the Giemsa-stained peripheral thick blood smear showed no haemoparasites but revealed fungal elements, specifically yeast cells. To confirm whether the findings represented contamination or true fungal sepsis, the test was repeated using a fresh capillary blood sample. The results were consistent with the initial findings, again showing the presence of yeast cells, as illustrated in Fig. 1 (a, b, c, d). However, fungal blood culture was not performed due to the lack of culture media.

**Fig. 2:**
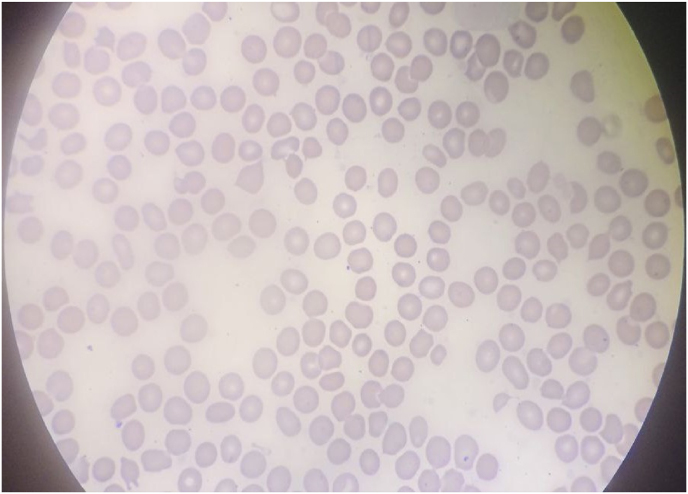
Peripheral thin blood smear with no yeast cells (Giemsa stain, 1000x magnification).

Based on these results, she was diagnosed with fungemia (fungal sepsis) and started on **oral fluconazole at a dose of 12 mg/kg/day, administered once daily as an oral suspension**. The total duration of antifungal treatment was **14 days.** By the 11th day of treatment with the antifungal agent, she showed significant improvement with the restoring of fever and cough. She continued both antifungal and anti-TB treatment with pyridoxine and was discharged in stable condition. Fifteen days after discharge, she returned for a follow-up visit, and all her physical measurements were within normal limits. Her CBC and BF were done and showing normal blood counts and no yeast cells in the peripheral blood smear (Fig. 2).

## 3 Discussion

Fungal pathogens contribute significantly to nosocomial infections, with *Candida* spp being the fourth most common group of pathogens isolated from patients in medical, surgical, and intensive care settings [7]. Invasive fungal infections, most commonly caused by *Candida*, account for nearly 70 % of all global invasive fungal infections, followed by *Cryptococcus* (20 %) and *Aspergillus* (10 %) [8]. However, other yeasts such as *Saccharomyces* and *Trichosporon* may also be implicated in bloodstream infections [1]. Since no further fungal identification was performed in this case, and “yeasts” could theoretically represent any of these genera, a diagnosis of fungemia due to yeast infection is more appropriate than attributing it specifically to *Candida*.

In the current case, the leukocyte count, particularly the lymphocyte count, was interpreted in light of the fungal load observed on peripheral smear. Similar findings have been reported in previous studies involving yeast infections [9,10]. It is proposed that yeast cells in the blood may interfere with automated hematology analyzers, causing potential comparison errors in the complete blood count (CBC), particularly by falsely elevating lymphocyte counts due to the morphological similarity between yeast forms and small lymphocytes. This represents an analytical challenge that underscores the importance of manual review. Therefore, laboratory technologists should carefully interpret CBC results, examining the white blood cell histogram and peripheral blood morphology before releasing final values.

Nevertheless, the increase in the infant's lymphocyte count in this case was not particularly marked and could be attributed to other common paediatric causes, such as viral infections or normal immune responses in infancy. This alternative explanation should be considered when interpreting lymphocytosis in such settings, alongside the possibility of interference by fungal elements [10].

Detection of yeast cells by blood smear review is uncommon, as it requires a high concentration of yeast in the blood. Branda et al. demonstrated that yeasts must be present at a concentration of at least 5 × 10^5^ CFU/mL to be visualized on a peripheral blood smear. Such a high fungal burden is rarely encountered, making the detection of yeasts in peripheral smears a noteworthy finding [11]. Peripheral smear examination remains a useful supplementary diagnostic tool in the early detection of yeast infections, especially in critically ill patients, when integrated with thorough reviews of CBC parameters and histograms. Repeated peripheral morphology assessments can further assist in distinguishing true yeast fungemia from possible smear contaminants.

A febrile patient presented with persistent symptoms despite multiple broad-spectrum antibiotics, a normal ESR, and negative tuberculosis results. On the 18th day of admission, yeasts were observed in the peripheral smear along with new-onset thrombocytopenia, a known finding in invasive yeast infections. Notably, this occurred after the patient had received approximately 28 days of antibiotic therapy (10 days at the referring facility and 18 days in our hospital). Prolonged exposure to broad-spectrum antibiotics is a well-documented risk factor for developing invasive yeast infections, likely disrupting normal flora and facilitating fungal overgrowth and translocation.

Although blood culture for fungal identification was not available, the consistent presence of yeast cells in repeated peripheral smears, together with clinical deterioration and thrombocytopenia, strongly supported the diagnosis of fungemia due to a yeast infection most likely *Candida* spp.

The overall clinical picture supports a complex disease course likely involving overlapping infections. It is probable that the patient initially had malaria and pneumonia, possibly complicated by nutritional rickets. The lack of clinical response to treatment and persistent fever led to empirical anti-TB therapy. The diagnosis of yeast fungemia, confirmed through repeated blood smears, appeared to represent a secondary nosocomial infection likely acquired or exacerbated during hospitalization due to prolonged broad-spectrum antibiotic use and clinical deterioration. The patient responded well to antifungal treatment with fluconazole, in addition to the ongoing anti-TB regimen, suggesting a multifactorial clinical course.

In conclusion, peripheral blood smear examination represents a practical and informative diagnostic aid for the early recognition of yeast infections, especially in resource-limited settings where advanced fungal testing is not readily available. Its timely application can facilitate prompt clinical decision-making and improve patient outcomes in challenging diagnostic scenarios.

## CRediT authorship contribution statement

**Yihun Bedaso:** Writing – review & editing, Writing – original draft, Investigation. **Tadesse Alemayehu:** Writing – review & editing, Writing – original draft, Investigation. **Amanuel Anegagregn Bizuneh:** Writing – review & editing, Writing – original draft, Investigation. **Dereje Zeleke Haile:** Writing – review & editing, Writing – original draft, Visualization, Investigation. **Seble Seifu Zeleke:** Writing – review & editing, Writing – original draft, Visualization, Investigation. **Mekdes Shifeta:** Writing – review & editing, Writing – original draft, Investigation. **Agete Tadewos Hirigo:** Writing – review & editing, Writing – original draft, Visualization, Methodology, Investigation, Formal analysis, Data curation, Conceptualization.

## Informed consent

Written informed consent was obtained from the patient's parent(s) for the publication of anonymized information in this article.

## Ethics approval

The institution does not mandate ethical approval for reporting individual cases and this study was exempt from IRB review. However, written informed consent was obtained from the patient's parent(s) for the publication of anonymized information in this article.

## Declaration of competing interest

The author(s) declare that there are no potential conflicts of interest concerning the research, authorship, and/or publication of this article.
